# Non-Pancreatic Digestive Enzymes

**DOI:** 10.3390/biom15091259

**Published:** 2025-08-30

**Authors:** Drucy Borowitz

**Affiliations:** Department of Pediatrics, Jacobs School of Medicine and Biomedical Sciences, University at Buffalo, Buffalo, NY 14203, USA; borowitz@buffalo.edu; Tel.: +1-(716)-323-0000; Fax: +1-(716)-323-0290

**Keywords:** amylase, lipase, protease, gastrin, maltase, sucrase, lactase, peptidases, malabsorption

## Abstract

Although the pancreas is the organ that produces the most critical digestive enzymes, there are other important contributors to the cleavage of food into absorbable units. Pre-pancreatic digestion of carbohydrates occurs through the action of salivary amylase. Pre-pancreatic digestion of fats is mediated by lingual and gastric lipases, and their action may be important as a signal for coordinated digestion. Pepsin, which is present in the stomach, initiates the digestion of dietary proteins into peptides and amplifies distal proteolysis. The major post-pancreatic intestinal carbohydrate-digesting enzymes are sucrase-isomaltase, maltase-glucoamylase and lactase-phlorizin hydrolase. There are no post-pancreatic mucosal enzymes that act on dietary triglycerides; however, the complete digestion of phospholipids depends on several brush border phospholipases. Intestinal processing is an important contributor to digestion of proteins, although mucosal proteases may serve as signaling proteins rather than as primary adjuncts to dietary protein digestion and absorption. This review describes the role of these non-pancreatic digestive enzymes in supporting nutritional health.

## 1. Overview

Digestion involves the enzymatic breakdown of dietary intake into components that can be absorbed and used for growth. The pancreas is the organ that produces the most critical enzymes, and individuals with exocrine pancreatic insufficiency have malabsorption because of loss of pancreatic secretions. These secretions neutralize gastric acid and provide the elements needed for the assimilation of dietary components. To prevent malnutrition, exocrine pancreatic insufficiency is managed by the provision of exogenous pancreatic enzyme replacement therapy. However, there are other important contributors to the cleavage of food into absorbable units. These other enzymes interact with substrates in the mouth and stomach prior to pancreatic secretion and along the intestinal brush border together with and following the action of pancreatic enzymes. Intestinal bacteria enzymes can also digest components of the diet and, in particular, may support colonic health [1]; however, this review will be limited to human intestinal enzymes. Perception of fats in the mouth increases pancreatic secretion and gastric digestion releases amino acids and free fatty acids. These control the gastric emptying rate and stimulate pancreatic enzyme secretion [2]. Post pancreatic digestion enables the process of digestion to be completed. This review will describe these human non-pancreatic digestive enzymes (Table 1), their actions, and how this intricate orchestration of the processing of food supports nutritional health.

## 2. Pre-Pancreatic Digestion

### 2.1. Pre-Pancreatic Digestion of Carbohydrates: Salivary Amylase

The mechanical digestion of food commences with the act of chewing, but enzymatic digestion also begins in the mouth. Saliva is secreted to wet food and enable it to be swallowed. Amylolysis by salivary amylase leads to a rapid decrease in glucose-polymer chain length and viscosity, facilitating the swallowing of food [3]. The products of α-amylase are dextrin fragments, maltose and isomaltose. Salivary and pancreatic amylases are similar but are encoded by different genes (AMY1 and AMY2, respectively). These amylases show different levels of activity against starches of various origins [4,5]. AMY1 is present at high levels in the parotid and lingual salivary glands. Human salivary amylase activity may have evolved as agriculture and harvesting of starches from grains and tubers supplemented hunting in our diets. The levels of salivary amylase in humans are by far the highest among primates [6]. Obligate meat-eaters, such as cats, never have salivary amylase.

Salivary amylase may have functions not directly related to its actions on the digestion of food. Data in rats shows that the AMY1 gene is expressed in taste glands, although at low levels, suggesting that it may have a role in food perception exclusive of the action of amylase on the texture of starches [7]. Others have suggested that humans can detect oligosaccharides independent of the sense of sweetness provided by glucose [8]. Salivary amylase also binds strongly to some oral streptococci, a function that may have a role in cariogenesis [9]. Although not related to its enzymatic activity, quantitation of salivary amylase has been used as an objective measure of behavioral response to stress as it reflects autonomic nervous system activity [10,11].

Salivary amylase is present and active in premature infants and levels appear to be stable across all age groups [12,13]. Although its amylolytic activity is small compared to that of pancreatic amylase, it may serve as compensation for lack of pancreatic amylase in patients with exocrine pancreatic insufficiency [14,15]. Encouraging extended mastication does not appear to be of benefit in increasing the amount of salivary amylase. Although studies vary in their conclusions, a systematic review of the literature concluded that “chewing in healthy people has minimum effect on the expression and activities of salivary proteins” [16].

### 2.2. Pre-Pancreatic Digestion of Fats

Most dietary fats are present as triglycerides found in meats, fish, dairy, oils, nuts and seeds. Pre-pancreatic enzymes that digest triglycerides are secreted in the mouth and stomach. Both lipases are active in an acid environment. Lingual and gastric lipases are present in the fetus from 26 weeks of gestation onwards. The activity of pre-pancreatic lipases may be a compensatory mechanism for digestion in patients with exocrine pancreatic insufficiency [17,18]. The coefficient of fat absorption in patients with exocrine pancreatic insufficiency who do not secrete pancreatic lipase is not zero, likely reflecting the activity of pre-pancreatic lipases [19,20,21,22].

#### 2.2.1. Lingual Lipase

Release of lingual lipase in the mouth is signaled by the autonomic nervous system following the start of a meal. The von Ebner’s glands on the surface of the tongue secrete lingual lipase in areas near fat taste receptors. A study in human neonates demonstrated a pH optimum of 3.5–6 and suggested that lingual lipase remains active in the fed stomach [23]. The action of lingual lipase releases non-esterified fatty acids (NEFA) from dietary fats. NEFA are signaling molecules. Although lingual lipase in rodents plays a key role in oral detection of fats, this effect does not seem to be as strong in humans [24]. Nonetheless, NEFA are released in humans in concentrations sufficient to depolarize taste receptor cells [25].

#### 2.2.2. Gastric Lipase

The proportion of lingual to gastric lipase varies among species. In the baboon and human, gastric lipase is present in much greater amounts than lingual lipase [26]. Lipolysis is greatest in the fundus of the stomach and is lowest in the antrum [27]. The biochemical properties of gastric lipase are the subject of an excellent review by Sams et al. [28]. Gastric lipase is stable and active in the pH range 2 to 7 with an optimal cleavage of fats at pH 4–5.4, the pH of the fed stomach. Under those conditions, 10–25% of acyl chains are released from dietary triglycerides [18,29]. Gastric lipase is resistant to degradation by pepsin. Like pancreatic lipase, the active site is controlled by a lid domain. Human gastric lipase is highly interactive with tri- and di-glycerides forming emulsions and has less activity on monoglycerides [30]. Species differences occur however gastric lipase cannot cleave phospholipids and cholesterol esters. Gastric lipase can hydrolyze all three ester bonds of triglycerides but preferentially cleaves the ester bond at the sn-3 position.

Another unique quality of human gastric lipase is that it changes the surface tension of droplets to allow bile salts to penetrate the phospholipid layers covering triglycerides. Unlike pancreatic lipase, gastric lipase is active in the presence of bile salts without needing a co-factor [31]. Human pancreatic lipase requires co-lipase, which stabilizes its active conformation, anchors it to the water–lipid interface, and displaces bile salts. Gastric lipase can initiate hydrolysis of milk triacylglycerol and thus is important for digestion of milk fat. Another pre-pancreatic lipase, bile-salt-stimulated lipase, is found in human milk. Activated bile salt-stimulated lipase cannot hydrolyze native milk fat globules. It is dependent on partial hydrolysis by gastric lipase, which triggers bile salt-stimulated lipase and pancreatic lipase activity [32].

Gastric lipolysis may be important as a signal for coordinated digestion as well as appetite and growth. Fatty acids, the products of gastric lipolysis, trigger the activity of pancreatic lipase [33]. The critical importance of gastric lipase is demonstrated in subjects who have undergone gastrectomy, whose regulation of pancreatic secretion becomes abnormal after gastric surgery, when gastric digestion and emptying are altered [34,35]. In addition, ghrelin is a gut hormone that aids the process of food uptake and growth hormone release and requires octanylation to be active [36]. Gastric lipase releases short and medium chain fatty acids such as octanoic acid that are specifically found at the sn-3 position of dairy products and various oils. It has been hypothesized that role of gastric lipase in releasing octanoic acid for ghrelin acylation is important for growth [28].

### 2.3. Pre-Pancreatic Digestion of Proteins: Pepsin

Dietary proteins are found in a range of foods including meat and fish as well as beans and nuts. Pepsin initiates the digestion of dietary proteins into peptides and amplifies distal proteolysis. In response to the hormone gastrin and to vagal stimulation, chief cells in the stomach secrete pepsinogen, an inactive zymogen that is converted to pepsin. Gastric acid is required to convert pepsinogen into pepsin. Pepsin is active at pH = 2, and transforms to an inactive conformation at pH 4.0–6.5 [37]. The digestive specificity of pepsin is broad, but the presence of phenylalanine or leucine increases the cleavage probability of porcine pepsin to greater than 40% and the presence of histidine, lysine, arginine or proline prohibits cleavage when found at the P1 position [38]. The strongest amino acid stimulants of Ca^2+^-sensing receptors, phenylalanine and tryptophan, are released by the action of pepsin [39]. Ca^2+^-sensing receptors control the release of gastrin and CCK from gastric acid-secreting parietal cells and neuroendocrine cells, thus increasing proteolysis [39,40]. Thus, pepsin both digests proteins directly and generates signals to aid digestion.

## 3. Post-Pancreatic Digestion

The intestinal brush border has microvilli that are the site for a wide range of digestive enzymes. These enzymes may be anchored to the brush border, and they can become incorporated into vesicles that are shed near the apices of microvilli. This helps nutrient hydrolysis to occur close to the absorptive surface [41].

### 3.1. Post-Pancreatic Digestion of Carbohydrates

Pancreatic α-amylase, which is more potent than salivary amylase, continues to digest the large oligosaccharides (α-dextrins) converting them to disaccharides (maltose), tri-saccharides (maltotriose), and oligosaccharides called limit dextrins which have four to nine glucosyl residues and an isomaltose branch. Alpha-amylase messenger RNA is found at high levels in the duodenum. This duodenal amylase appears to have a role in maintaining intestinal epithelial integrity and at high concentrations, inhibits glucose uptake by the brush border Na+/glucose cotransporter 1 (SGLT1), thus moderating high blood sugar swings from high dietary glucose loads [42]. Further digestion of the products released by amylase is mediated by other enzymes located on the intestinal epithelium. Trehalose, lactose and sucrose are not acted upon by amylase but there are specific enzymes in the small intestine to aid with digestion of these dietary components.

The major intestinal carbohydrate-digesting enzymes, including sucrase-isomaltase (SI), maltase-glucoamylase (MGAM) and lactase-phlorizin hydrolase are membrane bound and are not secreted. They require immediate contact with their substrates. As demonstrated by Amiri et al., respectively, they constitute 8.2%, 2.7% and 1.4% of total brush border membrane protein and show optimal activity at pH 6, with over 50% residual activity between pH 5 to pH 7 [43]. The substrates, their corresponding enzymes, and the subunits to which they are digested are in Table 2.

#### 3.1.1. Alpha Glucosidases

The name alpha-glucosidases describes four maltases, enzymes that convert linear starch into simple sugars. Two of these maltase activities are associated with sucrase-isomaltase (maltase Ib, maltase Ia). The other two are named maltase-glucoamylase (maltases II and III). The sucrase–isomaltase and maltase-glucoamylase complexes have similar structures and exhibit much sequence homogeneity. The protein is attached to the luminal membrane by a domain near the N-terminus. Complete digestion of α-linked glucose polymers to absorbable glucose takes place through the action of the 4 mucosal α-glucosidases in the small intestine.

##### Sucrase-Isomaltase

The sucrase-isomaltase (SI) complex has two subunits that digest different substrates. It is synthesized as a single polypeptide chain which is divided into two subunits, one of which remains attached to the small intestinal membrane. Eighty percent of the maltose that we eat is digested by this complex. Almost all the isomaltose formed by glucoamylase from limit dextrins is hydrolyzed by the SI complex. Levels of SI are highest in the jejunum and are lower in the duodenum and ileum [44].

Congenital sucrase-isomaltase deficiency (CSID), now called genetic SI deficiency, is the inability to digest sucrose and starch due to mutations in the SI gene that lead to no function. Maldigested sugars accumulate in the bowel leading to osmotic diarrhea, increased motility, and increased bacterial fermentation causing intestinal gas and bloating. Although classically it has been described as a disorder identified in infancy when solid foods and juices are added to the diet, it may present later in life in patients with symptoms similar to those seen in irritable bowel syndrome (IBS) [45]. These individuals have genetic variants that are milder than those seen in CSID. A diet low in carbohydrates that are poorly absorbed in the intestine is commonly used to control symptoms in patients with IBS, however it does not restrict sucrose (FODMAP diet; fermentable oligosaccharides, disaccharides, monosaccharides, and polyols). In one study, 50% of patients with diminished response to a low FODMAP diet were carriers of an SI variant that decreases SI enzymatic activity [46].

Identification of novel mutations has led to the categorization of genetic SI deficiency into three phenotypes based on the activity of the variants on protein trafficking, enzyme function and lipid raft association. Levels of enzyme activity can vary over a large range depending on the mutation [47]. Genetic SI deficiency occurs in 0.05–0.2% of individuals of European descent and approximately 5–10% among indigenous Greenlanders [48]. Inheritance is autosomal recessive and carrier frequencies have been reported as near 2% in non-Hispanic Whites, around 0.5% in Hispanic Whites and less than 0.05% in African Americans [48,49].

##### Maltase-Glucoamylase

The maltase-glucoamylase complex is like the SI complex but it has only one polypeptide chain. Maltase digests α-1,2- and α-1,3-disaccharides better than the other α-glucosidases. In addition, it can hydrolyze α-1,4 and α-1,6 linkages [50]. Amylase cleaves bonds within the interior of starch molecules. In contrast, glucoamylase is an exoglycosidase that begins enzymatic cleavage at the nonreducing end of a polysaccharide or limit dextrin, releasing glucose monosaccharides. It will digest a limit dextrin down to isomaltose, which can then be hydrolyzed by the sucrase–isomaltase complex. Maltase hydrolyzes small-sized malto-oligosaccharides to glucose; glucoamylase preferentially hydrolyzes longer malto-oligosaccharides. In humans, maltase activity increases along the intestine and reaches its highest activity in the distal ileum [44].

Although less common than SI deficiency, maltase-glucoamylase deficiency was documented by intestinal biopsy in 25% of 203 children with chronic abdominal pain [51]. These individuals had a normal gross appearance to the bowel, which is important because damage to the intestinal mucosa can cause secondary dissacharidase deficiencies. In a study by Viswanathan and Rao, 120 adult patients with unexplained gastrointestinal (GI) symptoms, 0.8% were maltase deficient, 9.2% of patients had deficiencies in all 4 α-glucosidases, and 0.8% had combined sucrase, maltase and isomaltase deficiency [52].

##### Trehalase

Trehalase is a brush border α-glycosidase. Trehalose is synthesized in many organisms where it may serve as a source of energy and carbon, but humans do not generate trehalose. Rather, the intestinal enzyme is present to digest this sugar, which is found primarily in mushrooms and baker’s yeast. A study in rats found trehalase mRNA expression to be highest in the duodenum, decreasing towards the distal ileum [53]. Isolated trehalase deficiency occurs in at least 8% of the Greenlanders but is extremely rare in intestinal biopsies of other populations [54,55].

#### 3.1.2. Lactase-Phlorizin Hydrolase

Lactose is not metabolized by amylase (which can only cleave α-glycolytic bonds) and thus enters the small intestine intact, where it is degraded by lactase-phlorizin hydrolase, commonly called lactase. Lactase is also termed β-glycosidase because it is the only brush border enzyme that hydrolyzes β- glycosidic bonds. Lactase acts on lactose and glycolipids to form glucose and galactose. Levels were found to be highest in human jejunum with decreasing activity towards the proximal and distal ends of the intestine [44].

The inability to digest lactose may be congenital, developmental, primary, or secondary. Congenital lactose intolerance is rare. It presents with severe diarrhea in the first days of life because of the complete inability to digest milk, the primary food source for infants [56,57]. Lactase activity increases during fetal development. Between 26 and 34 weeks of gestation, lactase activity is approximately one third of that found in full term babies. From 35 to 38 weeks of gestation, lactase activity increases to about 70% of term infants [58]. Thus, prematurely born infants have developmental lactose intolerance. Primary lactose intolerance results from decreased synthesis of lactase, either at the transcriptional or translational level [59]. As per the American Academy of Pediatrics, “Approximately 70% of the world’s population has primary lactase deficiency…In populations with a predominance of dairy foods in the diet, particularly northern European people, as few as 2% of the population has primary lactase deficiency. In contrast, the prevalence of primary lactase deficiency is 50% to 80% in Hispanic people, 60% to 80% in black and Ashkenazi Jewish people, and almost 100% in Asian and American Indian people… The age of onset and its prevalence differ among various populations. Approximately 20% of Hispanic, Asian, and black children younger than 5 years of age have evidence of lactase deficiency and lactose malabsorption, whereas white children typically do not develop symptoms of lactose intolerance until after 4 or 5 years of age.” [60]. Adults with unexplained GI symptoms may have lactose intolerance, either alone or in combination with deficiency of all four disaccharidases (“pan-disaccharidase deficiency”) [52]. Secondary lactase deficiency results when there is small intestinal injury with blunting of the intestinal villi and subsequent loss of lactase at their tips. Severe malnutrition, after enteric infections or with celiac disease, Crohn disease or other immune-related injury can lead to secondary lactase deficiency which improves when the intestinal mucosa has healed.

### 3.2. Post-Pancreatic Digestion of Fats

**Figure 1:**
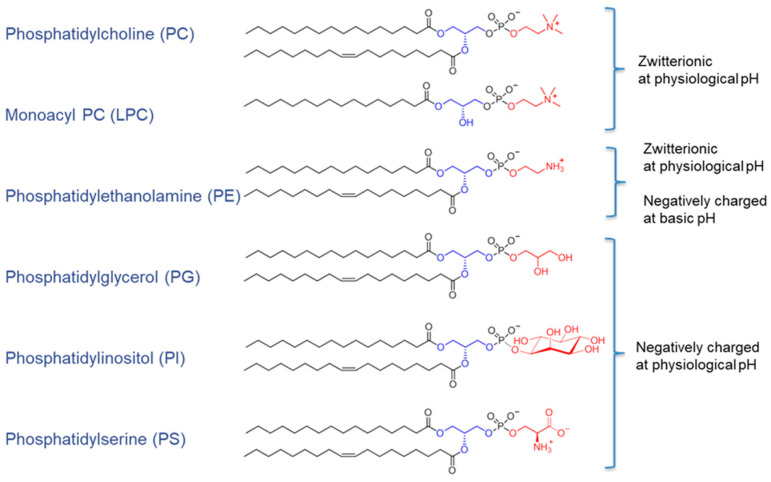
Structure of phospholipids. https://www.phospholipid-research-center.com/phospholipid/types/ (accessed on 29 August 2025). Reproduced with permission from van Hoogevest P et al., Eur. J. Lipid Sci. Technol. [61], Figure 2, published by Wiley-VCH GmbH 2021.

Although there are also cytoplasmic lipases, our focus is on brush border enzymes. The majority of lipid digestion and absorption occurs in the duodenum, primarily through the action of gastric and pancreatic lipases. The major dietary lipid class, triglycerides, are rapidly hydrolyzed by the combined action of gastric and pancreatic lipases in the stomach and upper small intestine. There are no post-pancreatic mucosal enzymes that act on triglycerides. However, the digestion of phospholipids is more extended. Phospholipids are found in foods such as eggs, organ meats, fish, shellfish, cereal grains, and oilseeds, as well as plant sources like soybeans, corn, and cruciferous vegetables. There are two types of phospholipids:

(1) Glycerophospholipids are key components of biological membranes. They have a hydrophobic tail that can become embedded in the cell’s membrane, connected by a glycerophosphate component to a hydrophilic head, which can be moieties such as choline, ethanolamine, or serine, among others (Figure 1) [61]. The pancreas assists in digestion of glycerophospholipids by secreting phospholipases A_1_ and A_2_ (PLA_1_ and PLA_2_, respectively) but studies in the PLA_2_ knock-out mouse suggest that distal, mucosal phospholipases play a major role in digestion and absorption of this class of lipids [62].

(2) Sphingophospholipids, such sphingomyelin and ceramide-1-phosphate, are important constituents of nervous system tissues. There are no pancreatic enzymes that digest sphingohospholipids.

In addition to glycerophospholipases and sphingophospholipases, there is a brush border-associated retinyl esterase that hydrolyzes sources of pre-formed vitamin A, mostly found in animal products such as liver, eggs, and dairy products.

#### 3.2.1. Glycerophospholipids

Phospholipase B_1_ (PLB): Pancreatic phospholipases A_1_ and A_2_ hydrolyze the ester bond at the sn-1 and sn-2 positions of glycerophospholipids, respectively. Mucosal PLB is a membrane-bound, calcium-independent enzyme with broad substrate specificity and a combination of both PLA_1_ and PLA_2_ activities, although it preferentially cleaves sn-2 ester bonds over sn-1 bonds. PLB plays an important role in digestion in the jejunum and ileum [63]. In addition, it may act as a retinyl esterase. Dietary retinol (Vitamin A) can be directly absorbed. Retinyl esters are fatty acids that are esterified to retinol. These cannot be absorbed until hydrolysis yields free retinol. The pancreas secretes retinyl esterases that have the greatest activity for esters whose fatty acyl portions are ≤10 carbons in length. Brush border retinyl esterase is active in the presence of bile salts and has its greatest activity toward the long-chain retinyl esters found in the diet [64]. The encoded protein is highly conserved.

#### 3.2.2. Sphingophospholipids

##### Alkaline Sphingomyelinase (Alk-SMase)

There are no pancreatic enzymes that hydrolyze sphingomyelin. Alk-SMase, the key enzyme responsible for SM hydrolysis, is present on the surface of the microvilli. It is released in active form into the intestinal lumen by bile salt and trypsin digestion [65]. It is protease resistant and acts in an alkaline environment. It is expressed neonatally. Decreased alk-SMase activity has been found in colon cancer and colitis [66,67].

##### Neutral Ceramidase

**Figure 2:**
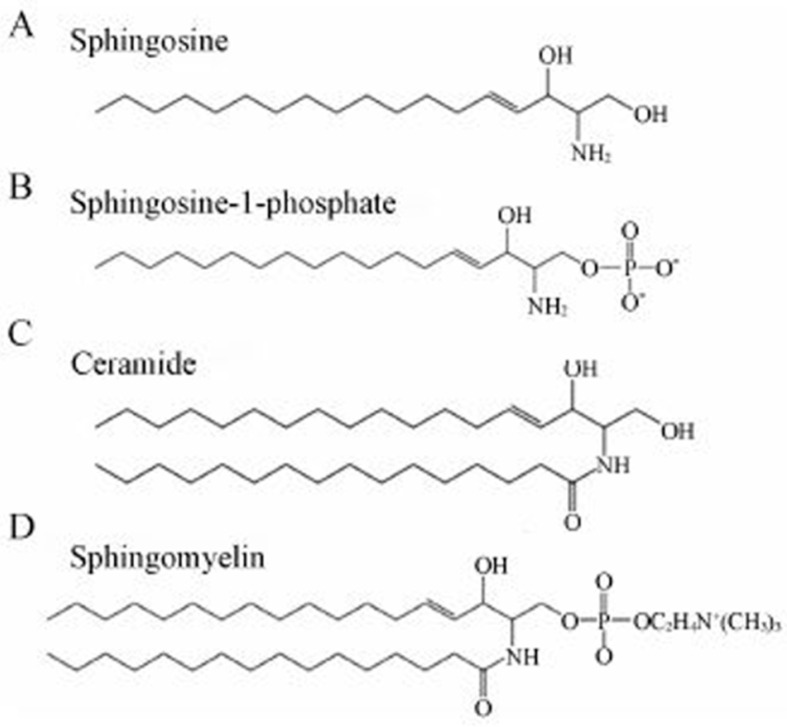
Structure of sphingophospholipids. https://www.creative-proteomics.com/blog/index.php/what-is-sphingomyelin/ (accessed on 29 August 2025). Reproduced with permission from Creative Proteomics 2025.

Ceramides are two-chained sphingolipids that are part of the structure of sphingomyelin. Thus, neutral ceramidase works in concert with Alk-SMase to hydrolyze ceramide to sphingosine and free fatty acids (Figure 2). Ceramide plays an important role in regulating cell proliferation and cell death and because of its role in complementing Alk-SMase it has also been implicated in the development of colon cancer. Data in animals suggests that neutral ceramidase activity is low in the proximal duodenum, increases to a plateau in the proximal jejunum and declines in the distal part of ileum. Some activity is also detectable in the colon [68]. Like alk-SMase it is stimulated by bile salts, is protease resistant, and is expressed neonatally.

### 3.3. Post-Pancreatic Digestion of Proteins

As with phospholipases, there are cytoplasmic peptidases, but our focus is on brush border enzymes. Brush border proteases are more correctly termed peptidases since they hydrolyze specific peptide bonds. Brush border enterokinase converts the proenzyme trypsinogen, which is secreted by the pancreas, to yield active trypsin. Enterokinase is a brush border peptidase but is not technically part of post-pancreatic digestion since it activates pancreatic protease. The activity of many of these brush border peptidases can be detected by 10–16 weeks of gestation and increases during fetal life. Many serve as signaling proteins rather than as primary adjuncts to dietary protein digestion and absorption. However, a study in rats demonstrated that 30% of ingested proteins were absorbed from isolated small intestinal loops following pancreatic duct ligation: larger amounts were absorbed if pepsin or pepsin plus acid were added to the lumen [69]. Thus, intestinal processing is an important contributor to digestion of proteins.

With the exception of pancreatic carboxypeptidases, pancreatic proteolytic enzymes are endopeptidases, cleaving peptide bonds within the polypeptide chain. Exopeptidases remove amino acids from the terminal ends of oligopeptides. Many brush border exopeptidases have been identified: what follows is a representative list.

#### 3.3.1. Aminopeptidase N

(APN/CD13) Aminopeptidase N (APN) is an N-terminal exopeptidase and is a membrane protein found in large amounts in the microvilli. In a pig model it represents more than half of the peptidase activity in the jejunum [70]. This zinc-dependent enzyme has three main functions: cleaving amino acids from the N-terminus of peptides, endocytosis (including of cholesterol), and signaling [71]. The digestive function of APN (and other brush border peptidases) is to convert proteins into oligopeptides and free amino acids that can subsequently be absorbed into the enterocyte. In addition, it cleaves a wide variety of metabolically active substrates such as enkephalins, kinins, and angiotensins, among others [71]. Enzyme activity increases in a proximal to distal manner in the small intestine and reaches maximum values in distal ileum [44].

#### 3.3.2. Dipeptidyl Aminopeptidase IV

(Dipeptidyl peptidase-4; DPP-4; CD26) enzymatically digests peptides, particularly those with alanine, proline, or serine residues in the penultimate position from the N-terminus. More than as an adjunct to incorporation of dietary protein for anabolism, aids in regulating metabolism and immune responses. DPP-4 helps degrade incretin hormones like glucagon-like peptide-1 (GLP-1) and glucose-dependent insulinotropic polypeptide (GIP), which are important for regulating blood sugar levels. Increased levels of GLP-1 lead to increased insulin and decreased glucagon production, which lowers blood glucose. DPP-4 inhibitors, also known as gliptins, are medications used to treat type 2 diabetes [72]. By blocking the action of DPP-4, levels of incretins increase, leading to lower blood glucose. Maximum activity is in the distal ileum [44].

#### 3.3.3. Glutamyl Aminopeptidase (ENPEP; Aminopeptidase A)

Glutamyl aminopeptidase is a zinc-dependent aminopeptidase that cleaves glutamatic and aspartatic acid residues from the N-terminus of polypeptides. In humans, ENPEP expression is highest in the terminal ileum [44,73].

#### 3.3.4. Carboxypeptidases

Carboxypeptidases remove amino acids from the C-terminal end of oligopeptides, rather than the N-terminal end. They are ubiquitous in the body and serve a wide range of functions in addition to digestion of dietary proteins. They also contribute to the regulation of blood fibrinolysis and the maturation of neuropeptides and hormones, among others. Pancreatic carboxypeptidases A1, A2 and B release a wide range of amino acids with a preference for those with small aliphatic and bulky aromatic side chains. However, pancreatic enzymes cannot release acidic amino acids from the C-terminus of polypeptides, such as glutamic or aspartic acid, even though these are among the most abundant amino acids in dietary proteins. Brush border carboxypeptidase O has this capacity and together with the action of the pancreatic carboxypeptidases, they can digest most proteins [74]. Carboxypeptidase activity is distributed throughout the small intestine [44].

#### 3.3.5. Angiotensin-Converting Enzymes (ACE and ACE2)

ACE is also a carboxypeptidase. It is present in the jejunal brush border where it aids in nitrogen absorption but likely plays a larger role in regulating local levels of signaling peptides. ACEs are present in many cells in the body. Vascular ACE is associated with the renin–angiotensin system, regulating peripheral blood pressure by converting angiotensin I to angiotensin II, inactivating bradykinin, and hydrolyzing enkephalins and other neuroactive peptides [75]. Epithelial intestinal ACE may act similar to its endothelial counterpart by controlling levels of endogenous bioactive peptides known to affect jejunal flows of sodium, chloride, bicarbonate and water. The GI renin–angiotensin system and its interactions with the GI microbiome are complex [76]. ACE2 has 40% overall identity to ACE and stabilizes neutral amino acid transporters. When not present, intestinal uptake of dietary amino acids such as tryptophan, is affected [77]. ACE2 is a SARS-CoV-2 receptor, and by down-regulating intestinal ACE2, SARS-CoV-2 could promote leaky gut syndrome, modify the gut microbiome and enhance systemic inflammation. As with DPP-4, the action of GI ACEs are less as true dietary digestive enzymes and more as regulators of gut homeostasis.

#### 3.3.6. A Few Endopeptidases Are Also Found in the Small Intestine

The most prevalent GI endopeptidase is neprilysin, which has broad substrate specificity. Intestinal neprilysin plays a role in regulating glucose homeostasis by inactivating incretins, including GLP-1. Meprin A is an endopeptidase that hydrolyzes peptides and insulin beta-chains [41].

## 4. Clinical Relevance

Digestion is a complex process and fortunately one with some redundancy. The most notable example is the compensation seen in patients with exocrine pancreatic insufficiency (EPI). EPI is seen in patients with cystic fibrosis, Schwachman-Diamond Syndrome, chronic pancreatitis, and pancreatic cancer. Although untreated EPI can lead to life-threatening malnutrition, it is also notable that malabsorption of fat and protein is diminished but not totally absent even in the absence of treatment, likely owing to the combined effects of pre- and post-pancreatic digestive enzymes. Clinicians often prescribe acid blocking medicines along with pancreatic enzyme replacement therapy for patients with EPI to overcome the inactivation of pancreatic lipase and protease by gastric acid. The natural stability of lingual and gastric lipase and of pepsin suggests that this strategy is not necessary to enhance pre-pancreatic digestion [23,37].

If there is structural or functional damage to the intestinal lining, brush border enzymes are reduced. This can be seen with severe diarrheal disease, celiac disease, small intestinal involvement in Crohn Disease or graft-versus-host intestinal damage and leads to nutrient malabsorption and consequent GI symptoms. The loss of brush border enzymes can lead to significant disruption of digestion. A congenital absence of brush border enterokinase leads to symptoms of protein malabsorption including failure to thrive and edema [78]. Infants with genetic sucrase-isomaltase deficiency present with osmotic diarrhea, distention and abdominal pain early in life, but partial expression of less severe genetic variants may not have overt symptoms until later in life [45]. As noted above, these genetic variants can be a cause of some symptoms of irritable bowel syndrome [46]. When the brush border is damaged there can also be deficient enteric hormone signaling of pancreatic secretion (secondary pancreatic insufficiency), so the presence of malabsorption symptoms seen with intestinal mucosal damage is multifactorial. Small intestinal bacterial overgrowth (SIBO) can be a cause of mucosal damage or can be a result of disruption of enteric integrity from any of the diseases noted above. SIBO can also be a consequence of dysmotility and is seen in patients with cystic fibrosis [79]. SIBO interferes with brush border enzyme activities through the action of bacterial proteases on the mucosa and leads to symptoms of malabsorption with similar pathophysiology to that seen with overt mucosal damage.

Short bowel syndrome (SBS), such as from congenital malformations or significant small intestine surgical resection, is the leading cause of intestinal failure in children [80] . The functional definition of SBS considers “the critical loss of the length of gut mass or its function below a minimum required for the adequate absorption of enteral nutrients and fluids to provide essential nourishment for the growth and the maintenance of life” [81]. Intestinal rehabilitation programs are designed to promote intestinal absorption. Controlling symptoms of carbohydrate malabsorption such as bloating or/and diarrhea resulting from loss of sucrase-isomaltase and lactase activity is important. Although not an explicit focus of current management, attention to the loss of non-pancreatic proteolytic enzymes, deserves attention. For example, loss of jejunum and thus activity of phospholipase B_1_ and neutral ceramidase can contribute to phospholipid malabsorption as well as that of other lipids and retinyl esters. Partial loss of the activity of DPP4 from distal ileal resection has the potential to lead to metabolic and immune system dysfunction. GLP-2 improves nutrient absorption and gut adaptation in rodents or humans with short bowel syndrome and has been proposed as a potential treatment [82]. Whether GLP-2 acts by increasing the activity of post-pancreatic digestive enzymes is an area for future exploration.

## 5. Conclusions

Although the pancreas is the organ that produces the most critical enzymes, there are other important contributors to the cleavage of food into absorbable units. These other enzymes interact with substrates in the mouth and stomach prior to pancreatic secretion and along the intestinal brush border in combination with and following the action of pancreatic enzymes. Awareness of these other components of digestion can be of help when assessing patients with GI symptoms such as bloating, abdominal pain or diarrhea and may be important when considering metabolic or nutritional deficiencies in patients with underlying intestinal disorders. Additionally, the efficacy of pancreatic enzyme replacement therapy cannot be assessed by improvement in symptoms alone. For example, ongoing bloating or abdominal pain may be caused by poor activity of post-pancreatic amylolytic enzymes. The aim of this review is to remind clinicians that digestion and absorption is a complex process and although important, it is not limited to the action of pancreatic enzymes.

## Figures and Tables

**Table 1:** Summary of non-pancreatic digestive enzymes.

	Carbohydrates	Fats	Proteins
Pre-pancreatic	Salivary amylase	Lingual lipase Gastric lipase	Pepsin
Post pancreatic	Sucrase-isomaltase Maltase-glucoamylase Trehelase Lactase	Phospholipase B1 Alkaline sphingomyelinase Neutral ceramidase	Aminopeptidase N Carboxypeptidase P Dipeptidyl aminopeptidase IV Glutamyl aminopeptidase Angiotensin-converting enzyme

**Table 2:** Digestion of carbohydrates.

	Enzyme	Carbohydrate Subunits	Bond Cleaved	Examples of Foods Providing Substrate
Products of amylase digestion:				
Isomaltose	Isomaltase	Glucose+glucose	α1,6	Wheat, cornmeal
Maltose	Maltase II	Glucose+glucose	Primarily α1,4 but also α-1,2, α-1,3 and α-1,6	
Limit dextrin	Glucoamylase	Glucose polymer with isomaltose branch	α1,4; cleaves from the non-reducing end	
Dietary disaccharides:				
Sucrose	Sucrase	Glucose+fructose	α1,2	Some fruits and vegetables
Trehalose	Trehelase	Glucose+glucose	α1,1	Mushrooms, baker’s yeast
Lactose	Lactase	Glucose+galactose	β1,4	Dairy products

## Data Availability

No new data were created or analyzed in this study. Data sharing is not applicable to this article.

## References

[1] Slavin J. (2013). Fiber and prebiotics: Mechanisms and health benefits. Nutrients.

[2] Fieker A., Philpott J., Armand M. (2011). Enzyme replacement therapy for pancreatic insufficiency: Present and future. Clin. Exp. Gastroenterol..

[3] Evans I.D., Haisman D.R., Elson E.L., Pasternak C., McConnaughey W.B. (1986). The effect of salivary amylase on the viscosity behaviour of gelatinised starch suspensions and the mechanical properties of gelatinised starch granules. J. Sci. Food Agric..

[4] Meites S., Rogols S. (1968). Serum amylases, isoenzymes, and pancreatitis. I. Effect of substrate variation. Clin. Chem..

[5] Hall F.F., Ratliff C.R., Hayakawa T., Culp T.W., Hightower N.C. (1970). Substrate differentiation of human pancreatic and salivary alpha-amylases. Am. J. Dig. Dis..

[6] Perry G.H., Dominy N.J., Claw K.G., Lee A.S., Fiegler H., Redon R., Werner J., Villanea F.A., Mountain J.L., Misra R. (2007). Diet and the evolution of human amylase gene copy number variation. Nat. Genet..

[7] Merigo F., Benati D., Cecchini M.P., Cristofoletti M., Osculati F., Sbarbati A. (2009). Amylase expression in taste receptor cells of rat circumvallate papillae. Cell Tissue Res..

[8] Lapis T.J., Penner M.H., Lim J. (2014). Evidence that humans can taste glucose polymers. Chem. Senses.

[9] Scannapieco F.A., Torres G., Levine M.J. (1993). Salivary alpha-amylase: Role in dental plaque and caries formation. Crit. Rev. Oral. Biol. Med..

[10] Nater U.M., Rohleder N. (2009). Salivary alpha-amylase as a non-invasive biomarker for the sympathetic nervous system: Current state of research. Psychoneuroendocrinology.

[11] Ali N., Nater U.M. (2020). Salivary Alpha-Amylase as a Biomarker of Stress in Behavioral Medicine. Int. J. Behav. Med..

[12] Murray R.D., Kerzner B., Sloan H.R., McClung H.J., Gilbert M., Ailabouni A. (1986). The contribution of salivary amylase to glucose polymer hydrolysis in premature infants. Pediatr. Res..

[13] Aguirre A., Levine M.J., Cohen R.E., Tabak L.A. (1987). Immunochemical quantitation of alpha-amylase and secretory IgA in parotid saliva from people of various ages. Arch. Oral. Biol..

[14] Skude G., Kollberg H. (1976). Serum isoamylases in cystic fibrosis. Acta Paediatr. Scand..

[15] Townes P.L., Moore W.D., White M.R. (1976). Amylase polymorphism: Studies of sera and duodenal aspirates in normal individuals and in cystic fibrosis. Am. J. Hum. Genet..

[16] Al-Manei K., Almotairy N., Bostanci N., Kumar A., Grigoriadis A. (2020). Effect of Chewing on the Expression of Salivary Protein Composition: A Systematic Review. Proteom. Clin. Appl..

[17] Abrams C.K., Hamosh M., Hubbard V.S., Dutta S.K., Hamosh P. (1984). Lingual lipase in cystic fibrosis. Quantitation of enzyme activity in the upper small intestine of patients with exocrine pancreatic insufficiency. J. Clin. Investig..

[18] Carriere F., Grandval P., Renou C., Palomba A., Prieri F., Giallo J., Henniges F., Sander-Struckmeier S., Laugier R. (2005). Quantitative study of digestive enzyme secretion and gastrointestinal lipolysis in chronic pancreatitis. Clin. Gastroenterol. Hepatol..

[19] Stern R.C., Eisenberg J.D., Wagener J.S., Ahrens R., Rock M., doPico G., Orenstein D.M. (2000). A comparison of the efficacy and tolerance of pancrelipase and placebo in the treatment of steatorrhea in cystic fibrosis patients with clinical exocrine pancreatic insufficiency. Am. J. Gastroenterol..

[20] Konstan M.W., Stern R.C., Trout J.R., Sherman J.M., Eigen H., Wagener J.S., Duggan C., Wohl M.E., Colin P. (2004). Ultrase MT12 and Ultrase MT20 in the treatment of exocrine pancreatic insufficiency in cystic fibrosis: Safety and efficacy. Aliment. Pharmacol. Ther..

[21] Wooldridge J.L., Heubi J.E., Amaro-Galvez R., Boas S.R., Blake K.V., Nasr S.Z., Chatfield B., McColley S.A., Woo M.S., Hardy K.A. (2009). EUR-1008 pancreatic enzyme replacement is safe and effective in patients with cystic fibrosis and pancreatic insufficiency. J. Cyst. Fibros..

[22] Trapnell B.C., Maguiness K., Graff G.R., Boyd D., Beckmann K., Caras S. (2009). Efficacy and safety of Creon 24,000 in subjects with exocrine pancreatic insufficiency due to cystic fibrosis. J. Cyst. Fibros..

[23] Liao T.H., Hamosh P., Hamosh M. (1984). Fat digestion by lingual lipase: Mechanism of lipolysis in the stomach and upper small intestine. Pediatr. Res..

[24] Kulkarni B.V., Mattes R.D. (2014). Lingual lipase activity in the orosensory detection of fat by humans. Am. J. Physiol. Regul. Integr. Comp. Physiol..

[25] Kulkarni B., Mattes R. (2013). Evidence for presence of nonesterified fatty acids as potential gustatory signaling molecules in humans. Chem. Senses.

[26] DeNigris S.J., Hamosh M., Kasbekar D.K., Lee T.C., Hamosh P. (1988). Lingual and gastric lipases: Species differences in the origin of prepancreatic digestive lipases and in the localization of gastric lipase. Biochim. Biophys. Acta.

[27] Abrams C.K., Hamosh M., Lee T.C., Ansher A.F., Collen M.J., Lewis J.H., Benjamin S.B., Hamosh P. (1988). Gastric lipase: Localization in the human stomach. Gastroenterology.

[28] Sams L., Paume J., Giallo J., Carriere F. (2016). Relevant pH and lipase for in vitro models of gastric digestion. Food Funct..

[29] Carriere F., Renou C., Ransac S., Lopez V., De Caro J., Ferrato F., De Caro A., Fleury A., Sanwald-Ducray P., Lengsfeld H. (2001). Inhibition of gastrointestinal lipolysis by Orlistat during digestion of test meals in healthy volunteers. Am. J. Physiol. Gastrointest. Liver Physiol..

[30] Fernandez S., Jannin V., Rodier J.D., Ritter N., Mahler B., Carriere F. (2007). Comparative study on digestive lipase activities on the self emulsifying excipient Labrasol, medium chain glycerides and PEG esters. Biochim. Biophys. Acta.

[31] Gargouri Y., Pieroni G., Lowe P.A., Sarda L., Verger R. (1986). Human gastric lipase. The effect of amphiphiles. Eur. J. Biochem..

[32] Bernback S., Blackberg L., Hernell O. (1990). The complete digestion of human milk triacylglycerol in vitro requires gastric lipase, pancreatic colipase-dependent lipase, and bile salt-stimulated lipase. J. Clin. Investig..

[33] Gargouri Y., Pieroni G., Riviere C., Lowe P.A., Sauniere J.F., Sarda L., Verger R. (1986). Importance of human gastric lipase for intestinal lipolysis: An in vitro study. Biochim. Biophys. Acta.

[34] MacGregor I.L., Martin P., Meyer J.H. (1977). Gastric emptying of solid food in normal man and after subtotal gastrectomy and truncal vagotomy with pyloroplasty. Gastroenterology.

[35] Mayer E.A., Thompson J.B., Jehn D., Reedy T., Elashoff J., Meyer J.H. (1982). Gastric emptying and sieving of solid food and pancreatic and biliary secretion after solid meals in patients with truncal vagotomy and antrectomy. Gastroenterology.

[36] Al Massadi O., Tschop M.H., Tong J. (2011). Ghrelin acylation and metabolic control. Peptides.

[37] Campos L.A., Sancho J. (2003). The active site of pepsin is formed in the intermediate conformation dominant at mildly acidic pH. FEBS Lett..

[38] Hamuro Y., Coales S.J., Molnar K.S., Tuske S.J., Morrow J.A. (2008). Specificity of immobilized porcine pepsin in H/D exchange compatible conditions. Rapid Commun. Mass. Spectrom..

[39] Conigrave A.D., Franks A.H., Brown E.M., Quinn S.J. (2002). L-amino acid sensing by the calcium-sensing receptor: A general mechanism for coupling protein and calcium metabolism?. Eur. J. Clin. Nutr..

[40] Taylor I.L., Byrne W.J., Christie D.L., Ament M.E., Walsh J.H. (1982). Effect of individual l-amino acids on gastric acid secretion and serum gastrin and pancreatic polypeptide release in humans. Gastroenterology.

[41] Hooton D., Lentle R., Monro J., Wickham M., Simpson R. (2015). The Secretion and Action of Brush Border Enzymes in the Mammalian Small Intestine. Rev. Physiol. Biochem. Pharmacol..

[42] Date K., Satoh A., Iida K., Ogawa H. (2015). Pancreatic alpha-Amylase Controls Glucose Assimilation by Duodenal Retrieval through N-Glycan-specific Binding, Endocytosis, and Degradation. J. Biol. Chem..

[43] Amiri M., Naim H.Y. (2017). Characterization of Mucosal Disaccharidases from Human Intestine. Nutrients.

[44] Skovbjerg H. (1981). Immunoelectrophoretic studies on human small intestinal brush border proteins--the longitudinal distribution of peptidases and disaccharidases. Clin. Chim. Acta.

[45] Danialifar T.F., Chumpitazi B.P., Mehta D.I., Di Lorenzo C. (2024). Genetic and acquired sucrase-isomaltase deficiency: A clinical review. J. Pediatr. Gastroenterol. Nutr..

[46] Zheng T., Eswaran S., Photenhauer A.L., Merchant J.L., Chey W.D., D’Amato M. (2020). Reduced efficacy of low FODMAPs diet in patients with IBS-D carrying sucrase-isomaltase (SI) hypomorphic variants. Gut.

[47] Gericke B., Amiri M., Scott C.R., Naim H.Y. (2017). Molecular pathogenicity of novel sucrase-isomaltase mutations found in congenital sucrase-isomaltase deficiency patients. Biochim. Biophys. Acta Mol. Basis Dis..

[48] Ellestad-Sayed J.J., Haworth J.C., Hildes J.A. (1978). Disaccharide malabsorption and dietary patterns in two Canadian Eskimo communities. Am. J. Clin. Nutr..

[49] Uhrich S., Wu Z., Huang J.Y., Scott C.R. (2012). Four mutations in the SI gene are responsible for the majority of clinical symptoms of CSID. J. Pediatr. Gastroenterol. Nutr..

[50] Lee B.H., Hamaker B.R. (2018). Maltase Has Most Versatile alpha-Hydrolytic Activity Among the Mucosal alpha-Glucosidases of the Small Intestine. J. Pediatr. Gastroenterol. Nutr..

[51] El-Chammas K., Williams S.E., Miranda A. (2017). Disaccharidase Deficiencies in Children with Chronic Abdominal Pain. JPEN J. Parenter. Enter. Nutr..

[52] Viswanathan L., Rao S.S. (2023). Intestinal Disaccharidase Deficiency in Adults: Evaluation and Treatment. Curr. Gastroenterol. Rep..

[53] Oesterreicher T.J., Nanthakumar N.N., Winston J.H., Henning S.J. (1998). Rat trehalase: cDNA cloning and mRNA expression in adult rat tissues and during intestinal ontogeny. Am. J. Physiol..

[54] Gudmand-Hoyer E., Fenger H.J., Skovbjerg H., Kern-Hansen P., Madsen P.R. (1988). Trehalase deficiency in Greenland. Scand. J. Gastroenterol..

[55] Murray I.A., Coupland K., Smith J.A., Ansell I.D., Long R.G. (2000). Intestinal trehalase activity in a UK population: Establishing a normal range and the effect of disease. Br. J. Nutr..

[56] Savilahti E., Launiala K., Kuitunen P. (1983). Congenital lactase deficiency. A clinical study on 16 patients. Arch. Dis. Child..

[57] Wanes D., Husein D.M., Naim H.Y. (2019). Congenital Lactase Deficiency: Mutations, Functional and Biochemical Implications, and Future Perspectives. Nutrients.

[58] Antonowicz I., Lebenthal E. (1977). Developmental pattern of small intestinal enterokinase and disaccharidase activities in the human fetus. Gastroenterology.

[59] Sterchi E.E., Mills P.R., Fransen J.A., Hauri H.P., Lentze M.J., Naim H.Y., Ginsel L., Bond J. (1990). Biogenesis of intestinal lactase-phlorizin hydrolase in adults with lactose intolerance. Evidence for reduced biosynthesis and slowed-down maturation in enterocytes. J. Clin. Investig..

[60] Heyman M.B., Committee on Nutrition (2006). Lactose intolerance in infants, children, and adolescents. Pediatrics.

[61] Van Hoogevest P., Tiemessen H., Metselaar J.M., Drescher S., Fahr A. (2021). The Use of Phospholipids to Make Pharmaceutical Form Line Extensions. Eur. J. Lipid Sci. Technol..

[62] Richmond B.L., Boileau A.C., Zheng S., Huggins K.W., Granholm N.A., Tso P., Hui D.Y. (2001). Compensatory phospholipid digestion is required for cholesterol absorption in pancreatic phospholipase A(2)-deficient mice. Gastroenterology.

[63] Nilsson A., Duan R.D. (2019). Pancreatic and mucosal enzymes in choline phospholipid digestion. Am. J. Physiol. Gastrointest. Liver Physiol..

[64] Rigtrup K.M., McEwen L.R., Said H.M., Ong D.E. (1994). Retinyl ester hydrolytic activity associated with human intestinal brush border membranes. Am. J. Clin. Nutr..

[65] Duan R.D., Cheng Y., Hansen G., Hertervig E., Liu J.J., Syk I., Sjostrom H., Nilsson A. (2003). Purification, localization, and expression of human intestinal alkaline sphingomyelinase. J. Lipid Res..

[66] Hertervig E., Nilsson A., Bjork J., Hultkrantz R., Duan R.D. (1999). Familial adenomatous polyposis is associated with a marked decrease in alkaline sphingomyelinase activity: A key factor to the unrestrained cell proliferation?. Br. J. Cancer.

[67] Sjoqvist U., Hertervig E., Nilsson A., Duan R.D., Ost A., Tribukait B., Lofberg R. (2002). Chronic colitis is associated with a reduction of mucosal alkaline sphingomyelinase activity. Inflamm. Bowel Dis..

[68] Lundgren P., Nilsson A., Duan R.D. (2001). Distribution and properties of neutral ceramidase activity in rat intestinal tract. Dig. Dis. Sci..

[69] Curtis K.J., Gaines H.D., Kim Y.S. (1978). Protein digestion and absorption in rats with pancreatic duct occlusion. Gastroenterology.

[70] Mamone G., Picariello G. (2023). Optimized extraction and large-scale proteomics of pig jejunum brush border membranes for use in in vitro digestion models. Food Res. Int..

[71] Mina-Osorio P. (2008). The moonlighting enzyme CD13: Old and new functions to target. Trends Mol. Med..

[72] Rohrborn D., Wronkowitz N., Eckel J. (2015). DPP4 in Diabetes. Front. Immunol..

[73] Holmes R.S., Spradling-Reeves K.D., Cox L.A. (2017). Mammalian Glutamyl Aminopeptidase Genes (ENPEP) and Proteins: Comparative Studies of a Major Contributor to Arterial Hypertension. J. Data Min. Genom. Proteom..

[74] Garcia-Guerrero M.C., Garcia-Pardo J., Berenguer E., Fernandez-Alvarez R., Barfi G.B., Lyons P.J., Aviles F.X., Huber R., Lorenzo J., Reverter D. (2018). Crystal structure and mechanism of human carboxypeptidase O: Insights into its specific activity for acidic residues. Proc. Natl. Acad. Sci. USA.

[75] Stevens B.R., Phillips M.I., Fernandez A. (1988). Ramipril inhibition of rabbit (*Oryctolagus cuniculus*) small intestinal brush border membrane angiotensin converting enzyme. Comp. Biochem. Physiol. C Comp. Pharmacol. Toxicol..

[76] Jaworska K., Koper M., Ufnal M. (2021). Gut microbiota and renin-angiotensin system: A complex interplay at local and systemic levels. Am. J. Physiol. Gastrointest. Liver Physiol..

[77] Penninger J.M., Grant M.B., Sung J.J.Y. (2021). The Role of Angiotensin Converting Enzyme 2 in Modulating Gut Microbiota, Intestinal Inflammation, and Coronavirus Infection. Gastroenterology.

[78] Holzinger A., Maier E.M., Buck C., Mayerhofer P.U., Kappler M., Haworth J.C., Moroz S.P., Hadorn H.B., Sadler J.E., Roscher A.A. (2002). Mutations in the proenteropeptidase gene are the molecular cause of congenital enteropeptidase deficiency. Am. J. Hum. Genet..

[79] Green N., Chan C., Ooi C.Y. (2024). The gastrointestinal microbiome, small bowel bacterial overgrowth, and microbiome modulators in cystic fibrosis. Pediatr. Pulmonol..

[80] Caporilli C., Gianni G., Grassi F., Esposito S. (2023). An Overview of Short-Bowel Syndrome in Pediatric Patients: Focus on Clinical Management and Prevention of Complications. Nutrients.

[81] Goulet O., Abi Nader E., Pigneur B., Lambe C. (2019). Short Bowel Syndrome as the Leading Cause of Intestinal Failure in Early Life: Some Insights into the Management. Pediatr. Gastroenterol. Hepatol. Nutr..

[82] Drucker D.J. (2001). Glucagon-like peptide 2. J. Clin. Endocrinol. Metab..

